# Decision vacuum and prepared delegation in cancer end-of-life decision-making: a scoping review

**DOI:** 10.1007/s00520-026-11394-3

**Published:** 2026-10-30

**Authors:** Seiichiro Kuroda, Yohei Iimura, Masako Kuroda

**Affiliations:** 1 Department of Pharmacy, The IMSUT Hospital, The Institute of Medical Science, The University of Tokyo, 4-6-1, Shirokanedai, Minato-ku, Tokyo 108-8639 Japan; 2 Kuroda Pharmacy, Hirai, Edogawa-ku, Tokyo Japan

**Keywords:** End-of-life decision-making, Decision vacuum, Prepared delegation, Decisional regret, Scoping review

## Abstract

**Context:**

End-of-life (EOL) decision-making profoundly shapes the quality of dying for advanced cancer survivors and the long-term psychological well-being (e.g., mitigating persistent decisional regret) of their bereaved families. While decision-making styles range from individual autonomy to clinician delegation across clinical and cultural contexts, their structural process determinants remain poorly synthesized.

**Objectives:**

To synthesize international (English-language) and Japanese evidence clarifying how EOL decision-making processes and timing influence psychological and economic outcomes in oncological care.

**Methods:**

Following PRISMA-ScR guidelines, we searched PubMed and Ichushi-Web for studies published from 2016 to 2026. Inclusion criteria focused on advanced cancer decision-making (involvement or delegation) and multi-dimensional outcomes, including psychological (satisfaction, regret, conflict), clinical (aggressiveness of care), and economic outcomes (financial toxicity, healthcare resource utilization). Narrative synthesis was performed on all 136 included studies (detailed matrix provided in Supplementary Table S1).

**Results:**

From 1,813 records, 136 studies were included. Physician-led decisions without prior value validation were associated with significantly higher decisional regret (*p* = − 0.400) and lower quality-of-life evaluations. A lack of preparedness contributed to a clinical “decision vacuum”—a state where unarticulated patient preferences lead to decision-making dictated by external system drivers or paternalism—frequently pushing vulnerable families toward non-evidence-based therapies, resulting in “Financial Toxicity” and inappropriate aggressiveness of care. Three key process moderators—validation of values, information transparency, and multidisciplinary coordination—were identified as essential for transforming delegation into a high-quality, value-concordant process (“prepared delegation”).

**Conclusion:**

EOL support must transcend the rigid dichotomy between individual autonomy and delegation. Implementing the “prepared delegation” model through early intervention and multidisciplinary validation can eliminate the “decision vacuum,” thereby preventing persistent decisional regret and protecting family economic and psychological well-being across the survivorship trajectory.

**Supplementary information:**

The online version contains supplementary material available at https://doi.org/10.1007/s00520-026-11394-3.

## Introduction

In contemporary palliative care and oncology, end-of-life (EOL) decision-making is a core process that defines the quality of care and facilitates life completion aligned with patients’ values [1, 2]. With the advances in medical technology, patients and their families are increasingly confronted with complex and ethical decisions, such as the selection of life-sustaining treatments and choices about care settings [3]. Proper decision-making in these scenarios not only preserves the patient’s dignity but has also been demonstrated to have long-term impacts on bereaved family members’ mental health and satisfaction [4, 5].

Against this background, advance care planning (ACP) has been internationally recommended as an essential foundation for facilitating communication among patients, families, and healthcare providers to support shared decision-making (SDM). However, the concepts and implementation of ACP are heavily dependent on medical systems and socio-cultural norms, and the limitations of applying a uniform model have been noted [3, 6, 7].

Decision-making styles exist along a broad spectrum. In much of the international literature, individual autonomy and ACP are emphasized, whereas discussions in Japan and other East Asian settings frequently highlight relational autonomy, family involvement, and trust-based delegation. Recent consensus studies on ACP in Asia underscore that family-centered decision-making and relational harmony are integral to local clinical realities, rather than mere barriers to autonomy [6, 7]. In Japan, the delegation style—often referred to as “omakase”—is frequently selected due to a desire to avoid burdening family members or a high degree of trust in clinicians [3, 8]. While such delegation can reduce the patient’s immediate psychological pressure, it carries the risk of decisions being driven by external circumstances if conducted without adequate dialogue or preparation.

In this study, we define a “decision vacuum” as a state in which explicit patient preferences are not articulated, resulting in decision-making being driven by external factors such as clinician judgment or systemic constraints. This vacuum is associated in the synthesized literature with downstream outcomes, including financial toxicity related to non-evidence-based therapies after the cessation of standard treatment [9, 10] and inappropriate medical-resource use, such as increased intensive care unit admissions and escalating medical costs [11, 12].

Previous discussions of EOL decision-making often contrast autonomy with delegation; however, this dichotomy may obscure the importance of process quality, including whether patient values are clarified, information is transparent, and families receive multidisciplinary support. Furthermore, even in Western settings, ACP implementation often faces barriers such as family conflict and prognostic uncertainty, suggesting that the challenges are not merely cultural but structural. Moreover, existing reviews are heavily biased toward English-language literature, and there is a lack of research that integrates Japanese-language or local evidence with international literature to clarify the clinical realities of non-Western EOL decision-making, including decision-making in Japan.

The objective of this scoping review is to synthesize evidence from both international literature (PubMed) and Japanese literature (Ichushi-Web) databases to systematize the determinants of psychological and economic outcomes in cancer end-of-life decision-making. This review clarifies how decision-making styles, specific topics, supportive interventions, and timing contribute to the satisfaction or psychological burden of patients and families. By integrating these dimensions, we aim to delineate the process-oriented determinates of high-quality decision-making and establish a clinically actionable framework that mitigates the “decision vacuum” across diverse healthcare contexts.

## Methods

### Design and guidelines

This study was conducted as a scoping review to systematically synthesize existing knowledge on end-of-life decision support and to construct an integrated framework for psychological outcomes. The review followed the PRISMA-ScR (Preferred Reporting Items for Systematic Reviews and Meta-Analyses extension for Scoping Reviews) guidelines to ensure transparent and rigorous reporting.

### Search strategy and information sources

To capture both international literature and Japanese clinical evidence, a systematic literature search was performed across two major databases: PubMed and Ichushi-Web (Japan Medical Abstracts Society database). This dual-database strategy was used to reduce reliance on English-language literature when examining Japanese EOL decision-making and to integrate Japanese evidence with broader international findings. The search period covered a 10-year span from April 2016 to March 2026, with the final search conducted in April 2026. The search strategy employed combinations of the following keywords:

- Palliative and end-of-life care: “terminal care,” “hospice care,” “palliative care,” “end of life.”
- Decision-making: “decision making,” “advance care planning (ACP),” “shared decision making (SDM),” “surrogate,” “proxy.”
- Psychological and cultural factors: “regret,” “satisfaction,” “conflict,” “burden,” “Japan,” “culture,” “omakase.”

The synthesis was designed to identify decision-making processes and outcome-related patterns across international and Japanese bodies of literature, rather than to conduct a formal country-by-country comparative analysis.

### Eligibility criteria

Studies were included based on the following criteria.

- Population: Adult patients with advanced cancer (oncology focus) and their family members or surrogate decision-makers.
- Content: Original research articles describing the decision-making process in end-of-life care.
- Core elements: Studies were required to include both of the following:
  - Element A, decision-making involvement: Descriptions of patient/family participation in decision-making or intentional delegation to others.
  - Element B, psychological outcomes: Specific evaluations of outcomes resulting from the decision, including psychological (satisfaction, regret, conflict, distress), clinical (aggressiveness of care), or economic outcomes (financial toxicity, healthcare resource utilization).
- Exclusion criteria: We excluded editorials, letters to the editor, case reports, and protocol papers without published results. General literature reviews were excluded. However, one Cochrane review was retained as an exception because it contained evidence directly relevant to interpersonal communication in EOL care, which was a central decision-support process examined in this review.

### Selection and screening process

A three-stage relevance screening process was used to ensure that included studies addressed both EOL decision-making involvement and psychological outcomes.

#### First stage, title screening

From 1813 records initially identified through database searching (PubMed: 1,136; Ichushi-Web: 677), 1076 records remained after duplicate removal and initial document-type screening. A total of 741 records were excluded because they did not match the target population or did not address EOL decision-making.

#### Second stage, abstract/summary screening

The remaining 335 records were assessed for the presence of both Element A and Element B; 105 records were excluded, primarily due to a lack of psychological outcome evaluations (*n* = 72).

#### Third stage (full-text/final summary assessment)

**Fig. 1:**
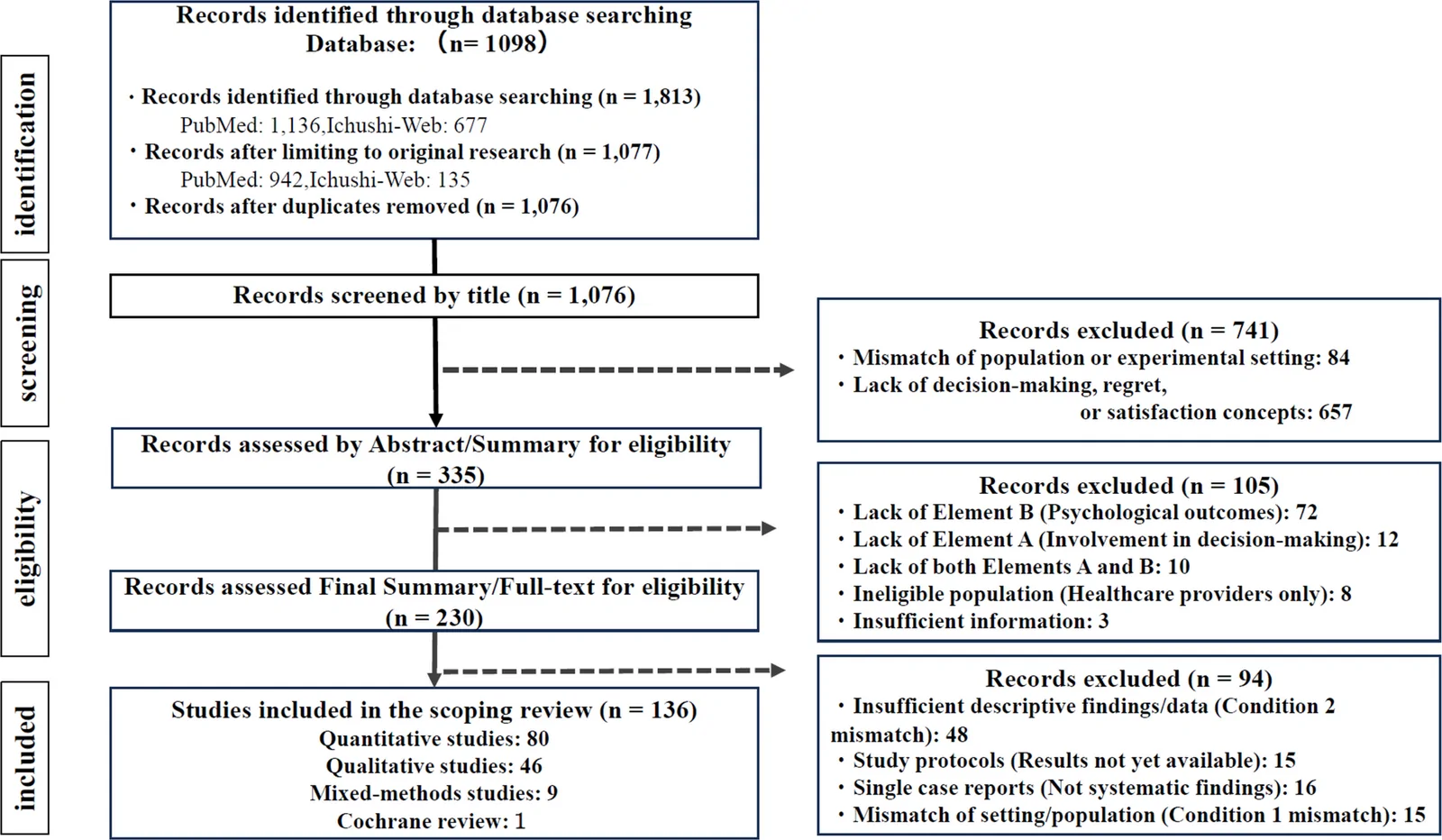
PRISMA flow diagram of the study selection process: A total of 1813 records were initially identified from PubMed and Ichushi-Web databases. After a three-stage relevance screening process based on decision-making involvement (Element A) and psychological outcomes (Element B), 136 studies were ultimately included in the narrative synthesis, comprising 80 quantitative studies, 46 qualitative studies, 9 mixed-methods studies, and 1 Cochrane review. The reasons for exclusion at each stage are detailed in the diagram

A total of 230 records underwent full-text or final summary assessment. At this stage, studies were retained if they met both final relevance conditions: they described a specific EOL decision-making scene, and they reported extractable statistical or qualitative findings related to psychological outcomes. These conditions were used to determine relevance to the review question, not to perform a formal quality or risk-of-bias assessment. Ultimately, 136 studies were selected for final analysis (Fig. 1). All 136 studies were included in the narrative synthesis; no smaller subset was analyzed separately. The complete matrix detailing the characteristics, decision contexts, and key outcomes of all 136 included studies is provided in Supplementary Table S1.

### Data extraction and synthesis

From the 136 included studies, we extracted fundamental characteristics, including publication year, study design, target population, and specific decision-making topics.

A narrative synthesis was then conducted, focusing on three major themes.

1. The impact of delegation (leaving decisions to others) on psychological outcomes.
2. Mechanisms by which SDM and active involvement contribute to reducing regret and increasing satisfaction.
3. Determinants of satisfaction rooted in the Process itself (e.g., information transparency, value validation) rather than just medical results.

This analytical approach allowed us to identify the mechanism of the “decision vacuum” caused by insufficient support and to construct the framework for “prepared delegation” as a culturally sensitive model for end-of-life care. Consistent with the purpose of a scoping review, which was to map and synthesize the available evidence rather than to grade the certainty of effects, we did not conduct a formal risk-of-bias or methodological-quality assessment for all included records.

## Results

### Study selection

From the 1,813 records initially identified through database searching, 1,076 records remained after removing duplicates and limiting the search to original research articles. Following the three-stage relevance screening process, a total of 136 studies were selected for narrative synthesis (Fig. 1). These included 80 quantitative studies, 46 qualitative studies, 9 mixed-methods studies, and 1 Cochrane review focused on interpersonal communication in EOL care [13].

### Characteristics of included studies

The 136 included studies were published between 2016 and 2026, with a significant surge in research activity from 2024 to 2026, accounting for 46 papers (33.8%). The research designs were predominantly quantitative (58.8%), though qualitative studies were also abundant (33.8%) [14]. Study populations were evenly distributed among patients (33.8%), family members/surrogates (33.8%), and patient-family dyads (32.4%). The primary decision-making topics were advance care planning (ACP) (24.3%) and end-of-life care (22.8%) [15] (Table 1).

**Table 1:** Characteristics of the 136 included studies. This table summarizes the foundational characteristics of the 136 studies included in this review, categorized by publication year, study design, target population, and specific decision-making topics. It highlights the recent surge in research activity (2024–2026) and a balanced distribution of focus between patients and family surrogates, reflecting the growing trend toward viewing end-of-life decision-making as a family-centered process

Characteristic	Category	Number (*n*)	Percentage (%)
1. Publication year	2016–2020	53	39.0%
	2021–2023	37	27.2%
	2024–2026*	46	33.8%
	(Total)	136	100.0%
2. Study design	Quantitative	80	58.8%
	Qualitative	46	33.8%
	Mixed methods	9	6.6%
	Others (e.g., cochrane review)	1	0.7%
3. Target population	Patients only	46	33.8%
	Family/surrogate decision-makers only	46	33.8%
	Both patients and families (including dyads)	44	32.4%
4. Decision-making topics	ACP/future preparedness	33	24.3%
	EOL care/palliative care (e.g., sedation, CPR)	31	22.8%
	Cancer treatment choice (e.g., chemotherapy)	15	11.0%
	Care setting/discharge planning	9	6.6%
	Others (e.g., SDM general, family meetings)	48	35.3%

^*^Data for 2026 is updated as of May 2026

### Decision-making styles and psychological outcomes

#### Impact of delegation and exclusion

Delegating decisions to physicians or being excluded from the process was strongly associated with negative psychological states. Specifically, physician-led decisions for care settings resulted in significantly higher decisional regret scores among bereaved families compared to shared decision-making (SDM) (*ρ* = − 0.400, *P* < 0.001) [16]. Furthermore, when physicians acted as the sole decision-makers, families’ evaluations of the patient’s Quality of Life (QOL) were significantly lower than in other styles (*P* = 0.014) [17]. Our synthesis of qualitative data revealed that families excluded from the process often felt like “pawns in an unclear pathway” [18, 19], with 71% expressing dissatisfaction due to the “lack of inclusion” [18].

#### Contribution of shared decision-making

Active participation significantly bolstered decisional certainty. A randomized trial of SDM for code status discussions demonstrated a reduction in decisional uncertainty by 7.06 points on the Decisional Conflict Scale [20]. Qualitatively, SDM fostered “post-bereavement self-growth”; families who made difficult choices together with patients later looked back on the caregiving experience as fulfilling, with some even stating, “it was fun” [14]. This commitment to the caregiving role through shared choice facilitated a sense of “life completion” and growth [21, 22].

#### Importance of process quality

The quality of the interaction—specifically the validation of values—governed long-term outcomes. Clinicians’ failure to validate what patients and families prioritized left survivors with a lasting “psychological weight” [5]. In contrast, a continuous relationship with a case manager or nurse who explored values allowed patients to feel “safe to entrust” their care, even when they did not seek full individual autonomy [23, 24].

### Determinants of satisfaction and regret by topic

#### Treatment selection

The communication style during the cessation of therapy was critical. In Japan, failing to explain the specific benefits of discontinuation (e.g., maintaining dignity and QOL) was a significant predictor of family dissatisfaction with the physician’s explanation (*β* = 0.228, *P* = 0.001) [25]. When families perceived EOL care as too aggressive (AOC), they reported significantly higher decisional regret (Cohen’s *d* = 0.49) [26]. Additionally, qualitative evidence indicates that approximately 40% of bereaved families experience regret regarding treatment cessation, which is primarily attributed to the quality of the decision-making process and the nature of their relationship with healthcare providers [27].

#### Choice of care setting

Discordance between preferred and actual place of death (POD) intensified depression and grief among bereaved families [28, 29]. A pivotal finding in the Japanese context was that the clinical guarantee of a “back-bed” (a secured hospital bed for emergency admission) served as a critical “turning point” that supported family peace of mind and the decision for home death [30].

#### End-of-life procedures

Regarding clinical procedures, *evidence from Japanese studies (Ichushi-Web)* highlighted that surrogate decisions regarding sedation or DNR when patient preferences were unknown caused intense ambivalence and guilt in 41% of cases. Conversely, *synthesized international studies (PubMed)* demonstrated that the “informed assent” approach successfully reduced surrogate psychological burden during code-status conversions [31] (Table 2).

**Table 2:** Factors associated with satisfaction and decisional regret by decision-making topic. This table categorizes the key determinants of psychological outcomes identified from the included studies, focused on three major clinical domains: (1) treatment choice, (2) selection of care settings, and (3) end-of-life clinical procedures. It distinguishes between factors that foster clinical consensus and satisfaction (e.g., prior validation of values, guarantee of a “back-bed”) and those that trigger long-term decisional regret (e.g., aggressiveness of care, discordance in preferences)

Decision-making topic	Factors leading to satisfaction/consensus	ID	Factors leading to regret/dissatisfaction	ID
1) Treatment choice	・Explanation of specific benefits of discontinuation (e.g., maintenance of QOL)	25 (Int), 27 (J)	・Aggressiveness of care (AOC) through inappropriate life-sustaining treatments	4 (Int), 26 (Int)
	・Sharing future quality of life (QOL) predictions	33 (Int)	・Neglect of emotional reactions to treatment side effects	34 (Int)
2) Choice of care setting	・Prior validation of patient values and preferences regarding the place of care	5 (J), 35 (Int)	・Discordance between preferred and actual place of death	28 (J), 29 (int)
	・Guarantee of a “back-bed” (secured hospital bed for emergency readmission)	30 (J)	・Pressured decisions driven by system constraints (e.g., discharge deadlines)	36 (J)
3) End-of-life procedures (e.g., sedation)	・Use of “informed assent” approach to reduce the psychological burden of active decision-making	32 (Int)	・Surrogate decisions made when the patient’s preferences are unknown	31 (J)
	・Application of shared decision-making (SDM) to clarify code status	20 (Int), 37 (Int)	・Physician-led (paternalistic) decisions excluding patients and families from the process	16 (Int), 17 (J)

^*^J Japanese literature (Ichushi-Web), *Int* International English literature (PubMed)

### Effective decision-support intervention methods

#### Digital and media interventions

Digital tools effectively reduced subjective uncertainty. The use of a video ACP tool significantly decreased decisional uncertainty from 28.3 to 21.7 (*P* = 0.02) [32] and was rated as “very helpful” by 85% of users [33]. Furthermore, mobile apps for recording consultations allowed 77% of patients to review information at home, which directly improved their knowledge and confidence in treatment selection [34].

#### Interpersonal and dialogue interventions

Structured communication led by nurses achieved remarkably high satisfaction. For instance, a specific emergency-department study on nurse-directed Goals-of-Care discussions reported 95–100% patient satisfaction scores [35]. Overall, multi-center international and Japanese data confirm that specialist palliative care team involvement significantly improves care satisfaction (SMD 0.36) and increases home death rates (OR 1.63) [36, 37].

#### Impact of timing

The timing of support determines the presence of the “decision vacuum.” Proactive, phased interventions from the early palliative stage fostered “future preparedness” (*r *= 0.38) [38, 39]. In contrast, decisions forced in “crisis phases”—such as immediately after an emergency admission—triggered confusion and long-term regret, as families were unprepared for the “sudden race to the end” [40, 41] (Table 3).

**Table 3:** Effective intervention methods and success factors in decision support. This table summarizes evidence-based intervention strategies classified into digital/media, interpersonal/dialogue, and mindfulness-based categories. It highlights the specific functional mechanisms of each approach—such as promoting information understanding and eliciting deep-seated values—and identifies the optimal clinical timing to maximize patient and caregiver preparedness throughout the illness trajectory

Intervention category	Specific methods	Key functions and timing	ID
1) Digital/media intervention	Video ACP tools, Web-based Decision Aids (DA), Consultation recording apps	**[Function]** Promoting information understanding, reducing decisional uncertainty	38 (Int), 39 (Int), 40 (Int)
		**[Timing]** From diagnosis to treatment selection (Early phase)	
2) Interpersonal/dialogue intervention	Nurse coaching, Multidisciplinary conferences, CNS coordination	**[Function]** Eliciting patient values, mediating intent-discordance within families	43 (int), 47 (J), 48 (J)
		**[Timing]** From symptom worsening to end-of-life (Mid-to-late phase)	
3) Mindfulness	Emotional control, reducing psychological barriers	**[Function]** Mitigating fear of death, psychological preparation for ACP	49 (Int)
		**[Timing]** Throughout the illness trajectory post-diagnosis	

^*^J Japanese literature (Ichushi-Web), *Int* International English literature (PubMed)

## Discussion

This scoping review synthesized findings from 136 studies to clarify how process quality influences psychological and economic outcomes in cancer EOL decision-making [13].

**Fig. 2:**
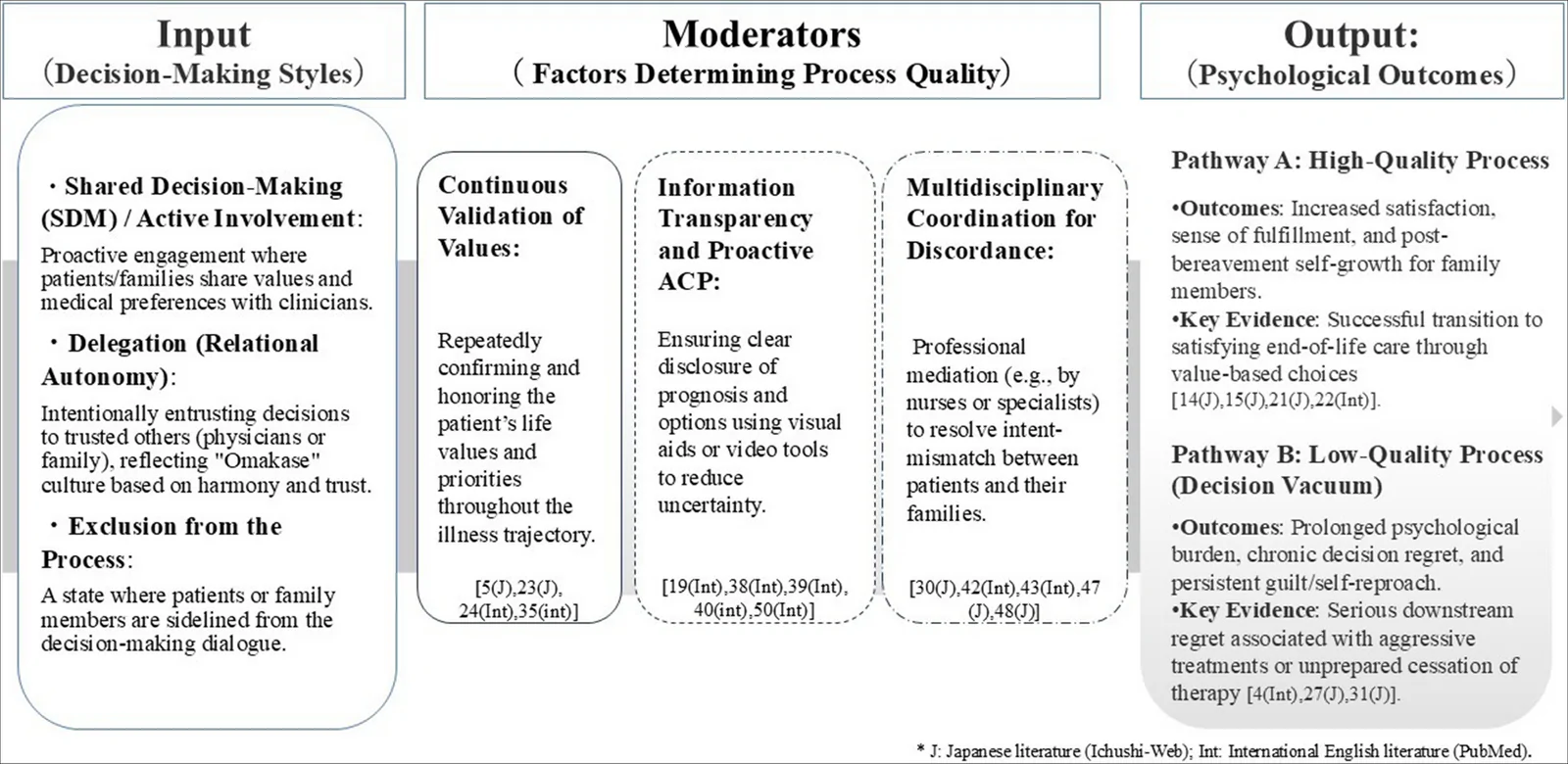
Conceptual model of decision support in end-of-life care: This model illustrates how the quality of the decision-making process—rather than the style of decision-making alone—determines psychological outcomes. Even in cases of Delegation (common in Japanese culture), positive outcomes such as high satisfaction and self-growth can be achieved if key moderators (Validation, Transparency, and Coordination) are functional. Conversely, a lack of these factors leads to a “Decision Vacuum,” which may contribute to long-term decisional regret and psychological burden

**Fig. 3:**
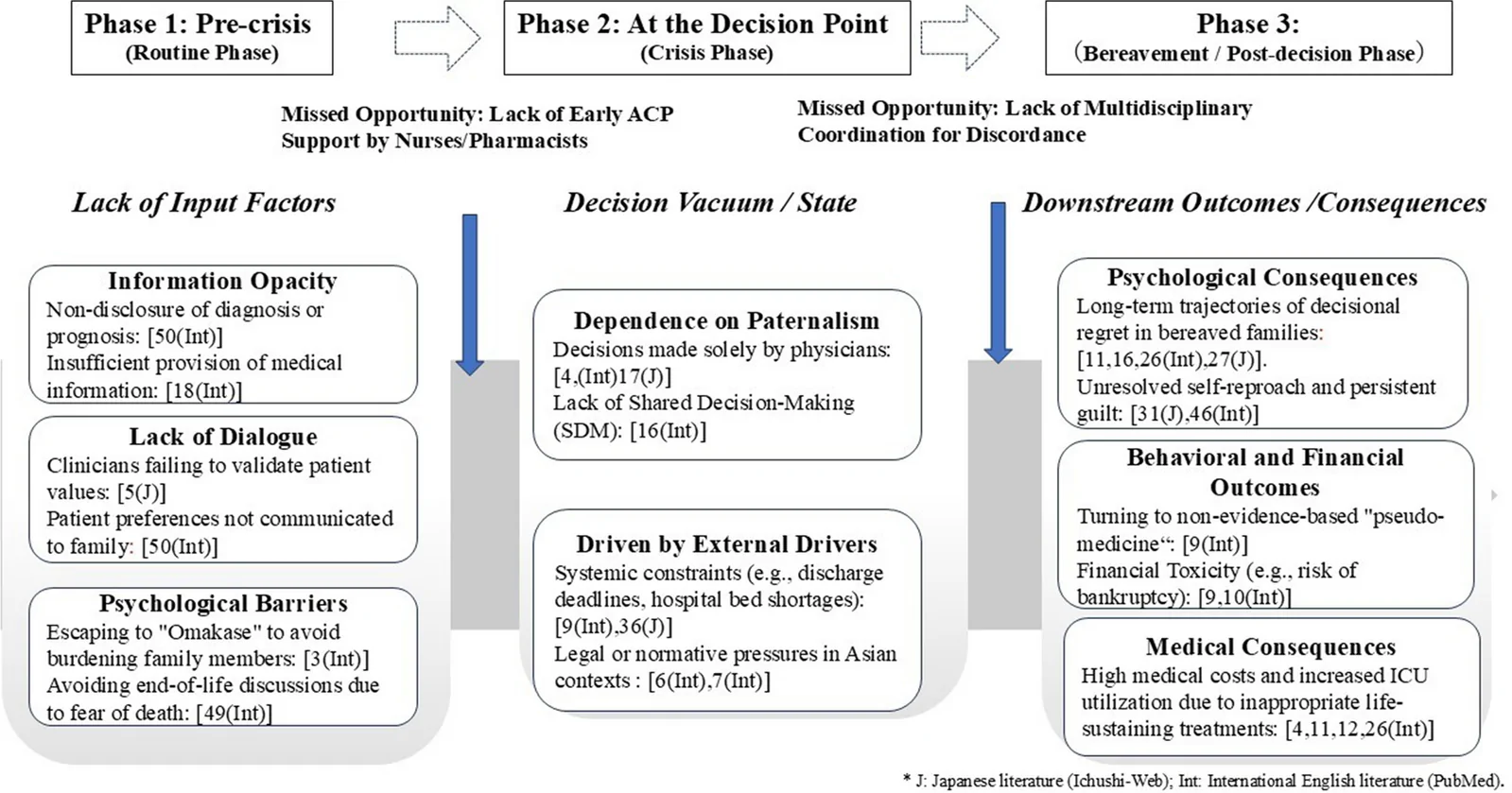
Conceptual model of unprepared decision-making: This figure illustrates the conceptual pathway from a lack of preparedness to negative clinical and psychological outcomes. In Phase 1, factors such as information opacity and lack of dialogue prevent the articulation of patient values. This leads to a Decision Vacuum in Phase 2, where decisions are driven by medical paternalism or systemic constraints rather than patient preferences. Finally, in Phase 3, this vacuum may contribute to Downstream Outcomes including long-term decisional regret, financial toxicity, and the inappropriate use of aggressive medical resources. The diagram emphasizes Missed Opportunities for early ACP and multidisciplinary coordination

A central contribution of this synthesis is the demonstration that the formal decision-making style—whether autonomy-based or delegation-based—does not intrinsically dictate outcome quality. Rather, our synthesis demonstrates that delegation itself is not inherently problematic. Negative outcomes (such as severe decisional regret, = − 0.400 [16], and downstream financial toxicity) arise when delegation occurs within an unprepared “decision vacuum,” where patient values remain unarticulated, and decisions are dictated by medical paternalism or system constraints (Figs. 2 and 3). Conversely, when delegation is preceded by value validation and trust-building (“prepared delegation”), it yields high satisfaction and low regret for both patients and bereaved surrogates.

### Clinical and economic harms of the “decision vacuum”

Our definition of the “decision vacuum”—a state in which external drivers such as hospital bed shortages or medical paternalism dictate care due to unarticulated patient values frames this absence of preparation as a clinically important process gap. The synthesized literature suggests that this vacuum is often exacerbated by a lack of accurate prognostic alignment [42] and unaddressed emotional responses to treatment-related outcomes [43], both of which are identified as significant contributors to downstream psychological, clinical, and economic burdens [9–12].

#### Behavioral and economic consequences

When standard therapy ceases without adequate preparation, families often lose their decisional anchors, leading to an increased reliance on non-evidence-based therapies [9]. This is not merely a medical choice but a manifestation of psychological vulnerability, often triggering financial toxicity that may threaten family economic stability during bereavement and after the patient’s death [9, 10].

#### Medical consequences

Surrogate decisions made without prior value validation frequently result in inappropriate aggressiveness of Care (AOC) [4, 26], characterized by high-intensity life-sustaining treatments. Our synthesis found that such decisions were associated with increased ICU utilization and escalating medical costs [11, 12], suggesting that a “decision vacuum” compromises both individual dignity and the equitable allocation of healthcare resources.

### Reconstructing Japanese-style ACP: “prepared delegation”

Across the included literature, delegation should not be interpreted only as passive abandonment of autonomy. In Japanese and East Asian contexts, delegation often reflects an active, relational choice rooted in trust and family harmony. Our synthesis strongly aligns with the recent Asian consensus on ACP, which emphasizes that advance care planning in Asian sectors must accommodate family-centered decision-making and relational autonomy [6, 7].

Crucially, this review demonstrates that barriers to EOL decision-making are not unique to Asian settings. Prognostic uncertainty, emotional avoidance, and family discordance frequently compromise ACP in Western settings where individual autonomy is prioritized [19, 44]. Therefore, the “prepared delegation” model—driven by continuous value validation, information transparency, and multidisciplinary coordination—offers a process-oriented framework. While particularly tailored to settings with relational delegation like Japan, it provides universal insights for transforming unsupported delegation into a value-concordant, high-quality decision process across diverse healthcare systems:

#### Continuous validation of values

Clinicians’ repeated confirmation of priorities serves as a “buffer” that mitigates the “psychological weight” and regret [5, 23, 24, 45].

#### Information transparency

Visualizing future options through video ACP tools improves subjective certainty, reducing uncertainty scores from 28.3 to 21.7 (*P* = 0.02) [32, 33, 46].

#### Multidisciplinary coordination

Professional mediation by nurses or specialists to resolve intent-discordance within families is essential to reach a consensus [30, 37, 47, 48].

“Prepared delegation” may provide a clinically useful framework for cancer EOL decision support in settings where patients and families prefer relational or delegated decision-making. Rather than serving as a universal standard, this model should be understood as a process-oriented approach that requires further evaluation across different diseases, care systems, and cultural contexts.

### Practical guidelines: informed assent and timing

#### Mitigating surrogate burden

Decisions regarding DNR or sedation when preferences are unknown often cause intense ambivalence and guilt, with families fearing they are “shortening the patient’s life” [41, 49]. The “informed assent” approach—where clinicians propose the best care and families authorize it—was found to be “acceptable” to most, effectively relieving families of the “weight of decision-making” [31]. This is further supported by findings that providing specific clinical recommendations, rather than leaving the decision entirely to families, is an acceptable communication style that significantly reduces the burden of surrogate decision-making in palliative care [50].

#### The importance of timing

While nurse-led dialogues achieved high satisfaction (95%–100%) due to timely implementation [35], decisions made in the “crisis phase”—such as immediately after an emergency admission—accelerated confusion and regret (*ρ* = − 0.400) [16]. In the Japanese oncology context, the clinical guarantee of a “back-bed,” or emergency readmission option, may provide the reassurance needed for some families to consider home-based care [30]. However, this support mechanism may not be available or function similarly in other health care systems, and its applicability should therefore be interpreted within the specific care context in which it was reported.

## Limitations

This study is subject to several limitations characteristic of its design. First, because this was a scoping review, we did not conduct a formal risk-of-bias or methodological-quality assessment of the 136 included studies. Therefore, the findings should be interpreted as a narrative mapping of available evidence rather than as a graded assessment of intervention effectiveness or evidence certainty. Second, although the inclusion of Ichushi-Web allowed the review to incorporate Japanese evidence and reduce reliance on English-language literature, the search was limited to PubMed and Ichushi-Web and may have excluded relevant studies indexed in other international or regional databases. In addition, many included studies described decision-making within individual countries or care settings rather than directly comparing Japan with other regions; therefore, cross-cultural interpretations should be understood as synthesized patterns rather than formal comparative conclusions. Third, the review focused primarily on oncology and cancer EOL decision-making. Therefore, the findings, including the proposed “prepared delegation” model, should be generalized cautiously to non-cancer illnesses, pediatric or non-oncology palliative care, acute-care decision-making, and health care systems with different family, legal, or institutional structures.

## Conclusion

In cancer EOL decision-making, decision support should move beyond a simple dichotomy between individual autonomy and delegation and instead focus on reducing the “decision vacuum” through improved process quality. The proposed “prepared delegation” model may help support value-concordant decision-making by emphasizing early preparation, information transparency, value validation, and multidisciplinary coordination.

## Supplementary information

Below is the link to the electronic supplementary material.

Supplementary file1 (XLSX 28 kb)

## Data Availability

No datasets were generated or analysed during the current study.
